# Management of B-cell lineage acute lymphoblastic leukemia: expert opinion from an Indian panel *via* Delphi consensus method

**DOI:** 10.3389/fonc.2023.1171568

**Published:** 2023-04-24

**Authors:** Vikram Mathews, Anu Korula, Anupam Chakrapani, Dinesh Bhurani, Jina Bhattacharyya, Manju Sengar, Pankaj Malhotra, Pavan Kumar Boyella, Pawan Kumar Singh, Prasanth Ganesan, Rishi Dhawan, Sameer Melinkeri, Sharat Damodar, Tuphan Kanti Dolai, Venkatraman Radhakrishnan

**Affiliations:** ^1^Department of Haematology, Christian Medical College, Vellore, Tamil Nadu, India; ^2^Department of Haematology, Christian Medical College, Vellore, Tamil Nadu, India; ^3^Clinical Hematology, Apollo Gleneagles Hospital, Kolkata, India; ^4^Department of Hemato-Oncology & Bone Marrow Transplant (BMT) Unit, Rajiv Gandhi Cancer Institute & Research Centre, New Delhi, India; ^5^Department of Clinical Hematology, Gauhati Medical College and Hospital, Guwahati, Assam, India; ^6^Medical Oncology Department, Tata Memorial Hospital, Mumbai, Maharashtra, India; ^7^Department of Clinical Hematology and Medical Oncology, Nehru Hospital, Postgraduate Institute of Medical Education & Research (PGIMER), Chandigarh, India; ^8^Department of Medical Oncology, Basavatarakam Indo American Cancer Hospital and Research Centre, Hyderabad, Telangana, India; ^9^Haemato-Oncology & Bone Marrow Transplant (BMT), B.L. Kapur (BLK)-Max Center for Bone Marrow Transplant, BLK-Max Superspeciality Hospital, New Delhi, India; ^10^Department of Medical Oncology, Jawaharlal Institute of Postgraduate Medical Education and Research (JIPMER), Puducherry, India; ^11^Clinical Hematology, All India Institute of Medical Sciences, Delhi, India; ^12^Department of Hematology, Deenanath Mangeshkar Hospital & Research Center, Pune, India; ^13^Mazumdar Shaw Medical Center, Narayana Health City, Bengaluru, Karnataka, India; ^14^Department of Haematology, Nil Ratan Sarkar (NRS) Medical College and Hospital, Kolkata, India; ^15^Department of Medical Oncology and Pediatric Oncology, Cancer Institute (WIA), Chennai, India

**Keywords:** B-cell acute lymphoblastic leukemia, relapsed/refractory, India, management, consensus, Delphi

## Abstract

**Introduction:**

Currently, there are no guidelines for the management of B-cell lineage acute lymphoblastic leukemia (B-ALL) from an Indian perspective. The diagnostic workup, monitoring, and treatment of B-ALL vary among different physicians and institutes.

**Objective:**

To develop evidence-based practical consensus recommendations for the management of B-ALL in Indian settings.

**Methods:**

Modified Delphi consensus methodology was considered to arrive at a consensus. An expert scientific committee of 15 experts from India constituted the panel. Clinically relevant questions belonging to three major domains were drafted for presentation and discussion: (i) diagnosis and risk assignment; (ii) frontline treatment; and (iii) choice of therapy (optimal vs. real-world practice) in relapsed/refractory (R/R) settings. The questionnaire was shared with the panel members through an online survey platform. The level of consensus was categorized into high (≥ 80%), moderate (60%–79%), and no consensus (< 60%). The process involved 2 rounds of discussion and 3 rounds of Delphi survey. The questions that received near or no consensus were discussed during virtual meetings (Delphi rounds 1 and 2). The final draft of the consensus was emailed to the panel for final review.

**Results:**

Experts recommended morphologic assessment of peripheral blood or bone marrow, flow cytometric immunophenotyping, and conventional cytogenetic analysis in the initial diagnostic workup. Berlin–Frankfurt–Münster (BFM)–based protocol is the preferred frontline therapy in pediatric and adolescent and young adult patients with B-ALL. BFM/German Multicenter Study Group for Adult Acute Lymphoblastic Leukemia–based regimen is suggested in adult patients with B-ALL. Immunotherapy (blinatumomab or inotuzumab ozogamicin) followed by allogeneic hematopoietic cell transplantation (allo-HCT) is the optimal choice of therapy that would yield the best outcomes if offered in the first salvage in patients with R/R B-ALL. In patients with financial constraints or prior allo-HCT (real-world practice) at first relapse, standard-intensive chemotherapy followed by allo-HCT may be considered. For subsequent relapses, chimeric antigen receptor T-cell therapy or palliative care was suggested as the optimal choice of therapy.

**Conclusion:**

This expert consensus will offer guidance to oncologists/clinicians on the management of B-ALL in Indian settings.

## 1 Introduction

Acute lymphoblastic leukemia (ALL) is a heterogeneous hematologic disorder characterized by the neoplastic proliferation of clonal precursor B or T cells in the bone marrow, peripheral blood, and extramedullary locations (1). B-cell lineage acute lymphoblastic leukemia (B-ALL) is the most common subtype of ALL, accounting for 85% of ALL cases (2). The survival outcomes for patients with ALL have improved substantially in the recent decade, especially among children primarily due to an increased understanding of pathogenesis and molecular genetics, the adoption of risk-stratified therapy, and the availability of newer treatment options (3, 4). A review by Arora et al. reported overall survival (OS) between 45% and 81% (follow-up: 4–5 years) in Indian children (median age: 5–10 years) with ALL (4). Radhakrishnan VS et al. reported a 5-year OS of 5.5%–58% and overall relapse rates between 24.3% and 57.1% (median time: 9–24 months) in adolescent and young adult (AYA) and adult patients (aged 10 years and above) with ALL (5). The monthly financial burden of childhood ALL has been reported to be 7.2 times the monthly per capita income of India (5). The burden of ALL in AYA patients appears to be even higher in India because India has a predominately younger patient population (5, 6). This also levies a substantial financial burden on a developing country like India due to the loss of productive years of both the patient and the caregiver, exorbitant treatment costs, and lack of comprehensive health insurance coverage (5). Further, laboratory evaluation of ALL is complex and often relies on advanced laboratory techniques, and financial challenges create significant problems in the timely delivery of treatment (5, 7). These often cause long interruptions or abandoning of treatment, often after successful initiation, which further leads to more resistant forms of the disease (8). It has been shown that intensification of treatment with combination therapies can lead to improvement in OS. However, the intensification of therapy also remains a significant challenge in India (8). This is due to limited resources to manage treatment-related adverse events, high prevalence of multidrug-resistant infections, and prolonged cytopenia with infections that further complicate cancer care (5, 8). Currently, there is a lack of consensus on the diagnostic workup and monitoring of B-ALL, and it varies among different physicians and institutes. In addition, there is a lack of consensus on the utility of different treatment options in frontline and relapsed/refractory (R/R) settings. In recent times, novel targeted immunotherapies, including monoclonal antibodies, antibody–drug conjugates, and cellular therapies, have shown significant promise in R/R adult B-ALL patients (9, 10). In lieu of the gaps identified, a countrywide consensus regarding protocols for diagnosis, treatment, and follow-ups that incorporate recent therapies is the need of the hour to improve treatment outcomes of B-ALL in India (5). Given the changing treatment landscape and the challenges faced in India, a panel of experts assembled to understand the current treatment scenario of B-ALL in India and reach a consensus regarding diagnostic and treatment approaches best suitable in the Indian setting. In this article, we have summarized expert opinions and recommendations on (i) diagnostic workup and risk assignment of B-ALL; (ii) frontline treatment of B-ALL; and (iii) choice of therapy in R/R B-ALL. Resource availability and cost constraints were considered while drafting consensus recommendations.

## 2 Methodology

### 2.1 Panel selection

**Figure 1:**
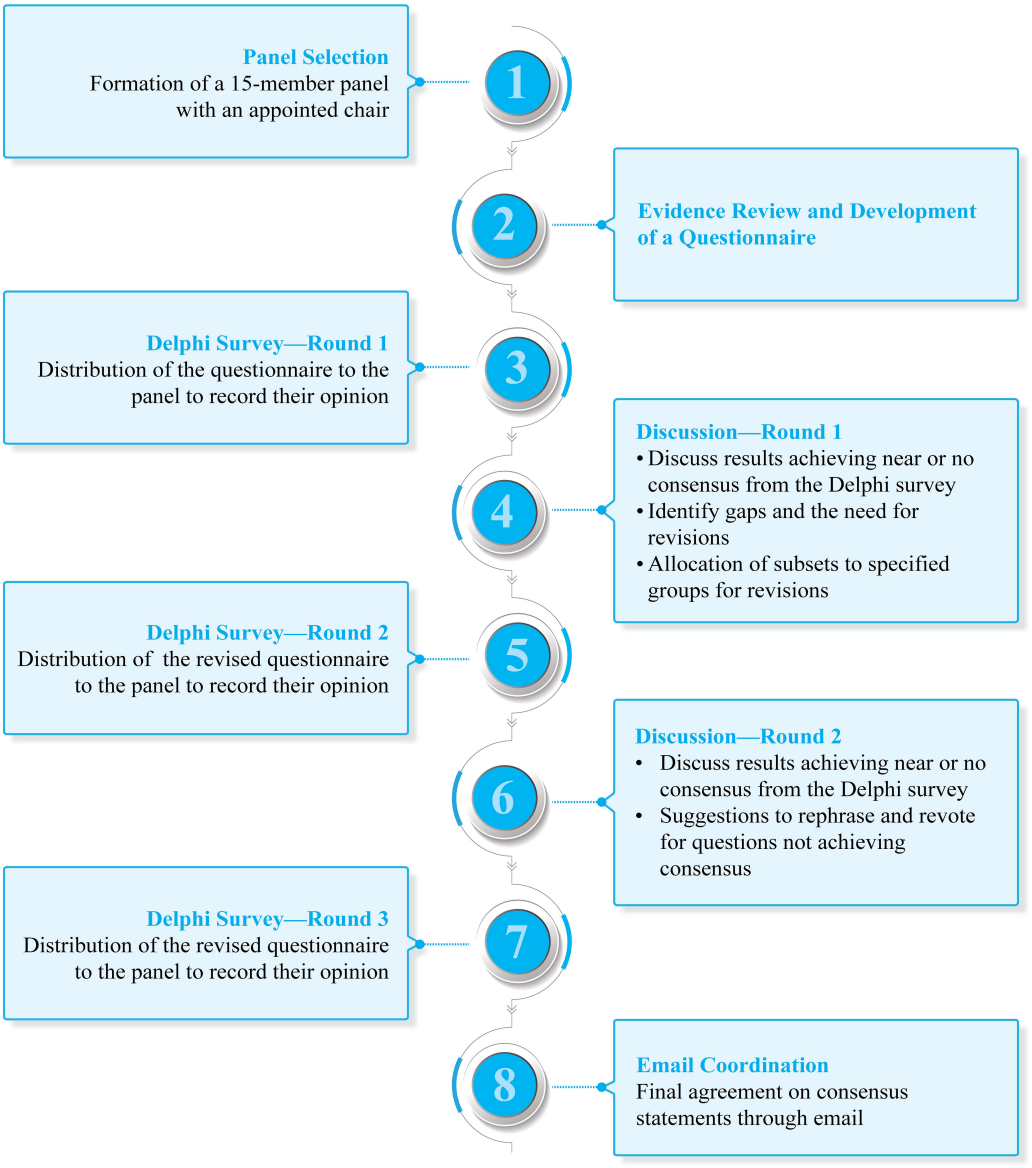
Overview of the consensus process used to create the clinical consensus statement.

A panel of 15 experts was selected (**Figure 1**) based on their academic track records and involvement in clinical research and experience in the field of B-ALL from various areas of the country (**Table S1** in **Supplementary Material**). A chair was identified among the panel members to drive the consensus process.

### 2.2 Evidence review

A literature review was carried out based on data from the PubMed database to identify relevant articles between January 2001 and September 2022 using keywords such as “B-cell acute lymphoblastic leukemia,” “diagnosis,” “management,” “relapsed/refractory,” and “guidelines.” The questionnaire was broadly segregated to include relevant questions under:

- Diagnosis and risk assignment
- Frontline treatment
- Choice of therapy in R/R settings (optimal and real-world practice)

*Defining optimal choice:* Optimal choice is the best possible option supported by evidence and is currently available in India, irrespective of cost or any other constraints. This should consider the absence of chimeric antigen receptor T-cell (CAR-T) therapy for second and subsequent relapse and in patients who had already undergone allogeneic hematopoietic cell transplantation (allo-HCT).

*Defining real-world practice:* Real-world choice is the best possible option currently available in India, keeping in mind cost and other constraints. This includes the option of a second allo-HCT in patients who have received an allo-HCT upfront or at first relapse.

The questionnaire was finalized in discussion with the chair and was rolled out to the panel members through an online survey platform (Delphi survey—round 1).

### 2.3 Consensus process

The experts discussed the results of the survey during a virtual expert panel meeting on April 22, 2022 (discussion—round 1). Modified Delphi consensus methodology was considered to arrive at a consensus (11). The level of consensus (**Table 1**) was categorized into high (≥ 80%), moderate (60%–79%), and no consensus (< 60%) (12). The differences in opinions were also discussed for modification of statements for the next round of voting (Delphi survey—round 2). The questions that received near or no consensus in the first round were discussed during the second meeting conducted virtually on August 6, 2022 (discussion—round 2). The recommendations were based on the responses to revised questions. The final round of voting was conducted to determine the definitive acceptance or rejection of a recommendation (Delphi survey—round 3). The final draft of the consensus was emailed to the panel for final review.

**Table 1:** Level of consensus.

**High**	When ≥ 80% of participants agree/strongly agree or disagree/strongly disagree with a statement.
**Moderate**	When 60%–79% of participants agree/strongly agree or disagree/strongly disagree with a statement.
**Low**	When < 60% of participants agree/strongly agree or disagree/strongly disagree with a statement.

Level of consensus: Adapted from: Jünger S et al., 2012 (12).

## 3 Results

The experts (N=15) analyzed evidence and guidelines on B-ALL management published between January 2001 and September 2022. Experts made their decisions based on the available evidence and their current practices in India. An effort was made to address optimal vs. real-world management of B-ALL based on loco-regional constraints. This article will first discuss the international guideline (the National Comprehensive Cancer Network [NCCN] and the European Society for Medical Oncology [ESMO]) recommendations followed by expert consensus.

### 3.1 Diagnostic workup and risk assignment of B-ALL

The diagnosis of ALL generally requires the demonstration of ≥ 20% bone marrow lymphoblasts upon hematopathologist’s review of bone marrow aspirate and biopsy materials (13–15). The NCCN and ESMO clinical practice guidelines recommend a comprehensive diagnostic approach in patients with ALL (13–15). This includes the following:

• Morphologic assessment of Wright–Giemsa–stained bone marrow aspirate smears, hematoxylin–eosin–stained core biopsy, and clot sections
• Immunophenotyping
  o Myeloperoxidase expression
  o B-lineage markers (CD19, CD79a, CD22, CD10, CD20, CD24, cIgM, and sIg [kappa or lambda])
  o T-lineage markers (CD3, CD1a, CD2, CD5, CD7, CD4, CD8, and TCR α/β or γ/δ)
• Cytogenetic analysis
• New genetics/genomics (gene expression profiling and next-generation sequencing [NGS])

The NCCN guideline also recommends a computed tomography (CT)/magnetic resonance imaging (MRI) scan of the head with contrast (in patients with major neurologic symptoms), testing for opportunistic infections, and an early allo-HCT evaluation at the time of initial diagnosis (14, 15). Optimal risk stratification and treatment planning require testing marrow or peripheral blood lymphoblasts for specific recurrent gene abnormalities using: (i) fluorescence *in situ* hybridization (FISH) for recurrent genetic abnormalities; (ii) reverse transcriptase-polymerase chain reaction (RT-PCR) testing for the detection of *BCR-ABL1* gene rearrangements, denoting an underlying t (9;22)(q34.1;q11.2)/*BCR-ABL1* chromosomal translocation typical of Philadelphia chromosome-positive (Ph+) ALL; and (iii) NGS for gene fusions and pathogenic mutations (13, 14).

The American Society of Clinical Oncology guidelines recommend testing for (7):

- t(12;21)(p13.2;q22.1) [*ETV6-RUNX1*]; t(9;22)(q34.1;q11.2) [*BCR-ABL1*]; t(v;11q23.3) [*KMT2A (MLL*) translocation]; iAMP21; and trisomy 4 and 10 in pediatric B-ALL.
- t(9;22)(q34.1;q11.2) [*BCR-ABL1*] and t(v;11q23.3) [*KMT2A (MLL*)] translocation in adult B-ALL.

***Consensus/recommendations on the diagnostic workup of B-ALL***

**Figure 2:**
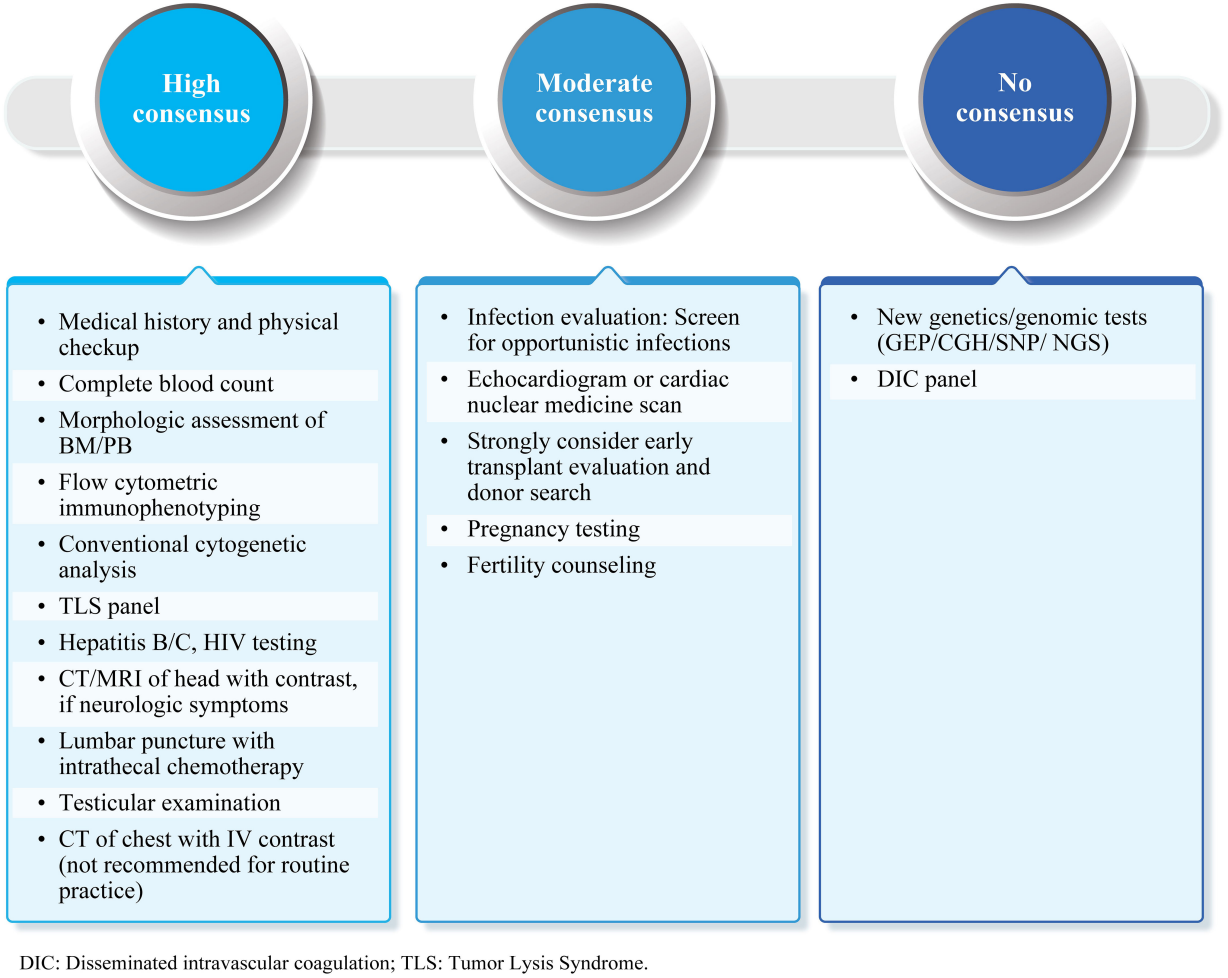
Initial diagnostic workup: Summary of expert consensus/recommendations. B-ALL, B-cell lineage acute lymphoblastic leukemia; BM, Bone marrow; PB, Peripheral blood; CT, Computed tomography; MRI, Magnetic resonance imaging; IV, Intravenous; NGS, Next-generation sequencing; CGH, Comparative genomic hybridization; SNP, Single-nucleotide polymorphism; GEP, Gene expression profiling; TLS, Tumor lysis syndrome; DIC, Disseminated intravascular coagulation.

The initial workup for B-ALL patients should include an evaluation of medical history and physical examination, along with laboratory and imaging studies (**Figure 2**). Experts recommended complete blood count, morphologic assessment of peripheral blood or bone marrow, flow cytometric immunophenotyping, and conventional cytogenetic analysis in the initial diagnostic workup (high consensus). A minimum panel of markers that includes CD19 plus CD22 for B-ALL is suggested (high consensus). Other recommended tests include hepatitis B/C and HIV evaluations. Female patients in reproductive age may undergo pregnancy testing (moderate consensus), and all male patients should be evaluated for testicular involvement of disease (high consensus). Experts suggested a CT/MRI scan of the head with contrast to detect meningeal disease, chloromas, or central nervous system (CNS) bleeding for patients with major neurologic symptoms at diagnosis. CNS involvement should be evaluated through lumbar puncture at the time of initial scheduled intrathecal therapy (high consensus). Assessment of cardiac function is important for patients with prior cardiac history, cardiac dysfunction, and elderly patients (moderate consensus). Screening for opportunistic infections, early allo-HCT evaluation, and donor search should be considered (moderate consensus).

Risk-directed treatment is an essential aspect of B-ALL management. Thus, it is important to assign risk categories to patients to ensure appropriate treatment decisions. The assignment of risk categories is primarily dependent on the availability of resources. The expert panel group at the 2013 Asian Oncology Summit proposed a four-tier system (basic, limited, enhanced, and maximum) based on which recommendations could be developed (16). In the case of basic resource settings, risk assignment can be based on age, presenting leukocyte count, and early treatment response as assessed by peripheral blood blast cell count. Additional molecular and cytogenetic features can be evaluated with the availability of enhanced resources (16). This stratification was modified and adapted to the Indian setting to evaluate the experts’ opinions (**Table 2**).

**Table 2:** Risk assignment stratification of B-ALL.

Risk assignment level	Criteria
1​	Age, leukocyte count, day 8 peripheral blood response​
2​	Age, leukocyte count, immunophenotype (T cell vs. B cell), prednisone response or day 8 peripheral blood or bone marrow response, end of induction bone marrow response. If available, RT-PCR for *BCR-ABL1*, cytogenetics for Philadelphia chromosome, or FISH for *BCR-ABL1*​
3​	RT-PCR for *BCR-ABL1* and *MLL-AFF1*, cytogenetics for hyperdiploid > 50, FISH for *BCR-ABL1*, and flow cytometry for MRD measurements
4​	*ABL*-kinase domain mutation analysis, especially the T315I mutation for selection of alternative tyrosine kinase inhibitors, pharmacogenetics,​ NGS for IgH/TCR rearrangements

B-ALL, B-cell lineage acute lymphoblastic leukemia; MRD, Minimal residual disease; RT-PCR, Real-time reverse transcription-polymerase chain reaction; FISH, Fluorescence in situ hybridization; IgH: Immunoglobulin heavy chain; TCR, T-cell receptor; NGS, Next-generation sequencing.

***Consensus/recommendations on risk assignment criteria***

Experts recommended the following risk assignment criteria best suitable in Indian settings (levels 2 and 3; moderate consensus):

- Age, leukocyte count, immunophenotype (T cell vs. B cell), prednisone response or day 8 peripheral blood or bone marrow response, end of induction bone marrow response. If available, RT-PCR for *BCR-ABL1*, cytogenetics for Philadelphia chromosome, or FISH for *BCR-ABL1*
- RT-PCR for *BCR-ABL1 and MLL-AFF1*, cytogenetics for hyperdiploidy > 50, FISH for *BCR-ABL1*, and flow cytometry for MRD measurements

### 3.2 Frontline treatment of B-ALL

The ESMO recommends age stratification for appropriate treatment of ALL as the treatment outcome of ALL is often age-associated (AYA: 15/18 to 35/40 years; adults: 35/40 to ≤ 55/60 years; elderly: above 55/60 years), hence necessitating age-based protocols (13). In India, currently there is a lack of consensus regarding age thresholds to categorize pediatric, AYA, and adult ALL (5). Various clinical trials have evaluated the efficacy and safety of chemotherapy regimens (Berlin–Frankfurt–Münster [BFM], Multicenter protocol 841 [MCP-841], Children’s Oncology Group [COG], United Kingdom Acute Lymphoblastic Leukemia [UKALL]) in the front line in children with ALL (15). Adapting these protocols in Indian settings has improved patient outcomes in the last decade; however, treatment-related mortality (11%–25%) and disease relapse (relapse rates: 15%–41%) have been reported in children in Indian settings (17–19). In 2013, the Indian Collaborative Childhood Leukaemia group (ICiCLe) developed a risk-stratified treatment protocol for the management of first presentation ALL based on cytogenetics and MRD levels (at the end of induction) in children (aged: 1–18 years) (20). Initial risk classification was based on lymphoblast lineage, age, leucocyte count, disease bulk, CNS disease status, leukaemia cytogenetics and prednisolone response at treatment day 8. The final risk stratification was determined at the end of the induction treatment phase and was based on treatment response, including remission status and the level of bone marrow MRD (20). The protocol is specific to Indian patients with ALL and is designed to (i) decrease toxicity and mortality in induction by shortening the duration of prednisolone therapy in patients with non–high-risk ALL and (ii) improve event-free survival in risk groups by replacing doxorubicin with mitoxantrone in delayed intensification (20). In India, treatment protocols used in AYA and adult ALL include MCP-841, BFM-90, chemotherapy plus tyrosine kinase inhibitor (TKI), German Multicenter Study Group for Adult Acute Lymphoblastic Leukemia (GMALL), and hyper-cyclophosphamide, vincristine, doxorubicin, dexamethasone (hyper-CVAD) (5). A real-world study by Malhotra P et al. found that a modified BFM regimen in adult ALL patients (> 12 years) in resource-limited settings resulted in complete remission (CR) of 85.6% after induction (5-year event-free survival: 21.6%) (21). A retrospective study was done on Indian adult ALL patients, which showed a 5-year OS of 38% and a CR rate of 82.2% with a modified GMALL regimen (8, 22). A more recent report from the Indian Acute Leukaemia Research Database and Hematology Cancer Consortium highlighted that BFM protocol (BFM-90, BFM-95, or BFM-2000) was the most common regimen used in AYA patients (aged 15–29 years) with ALL (23).

***Consensus/recommendations on frontline treatment of B-ALL***

**Figure 3:**
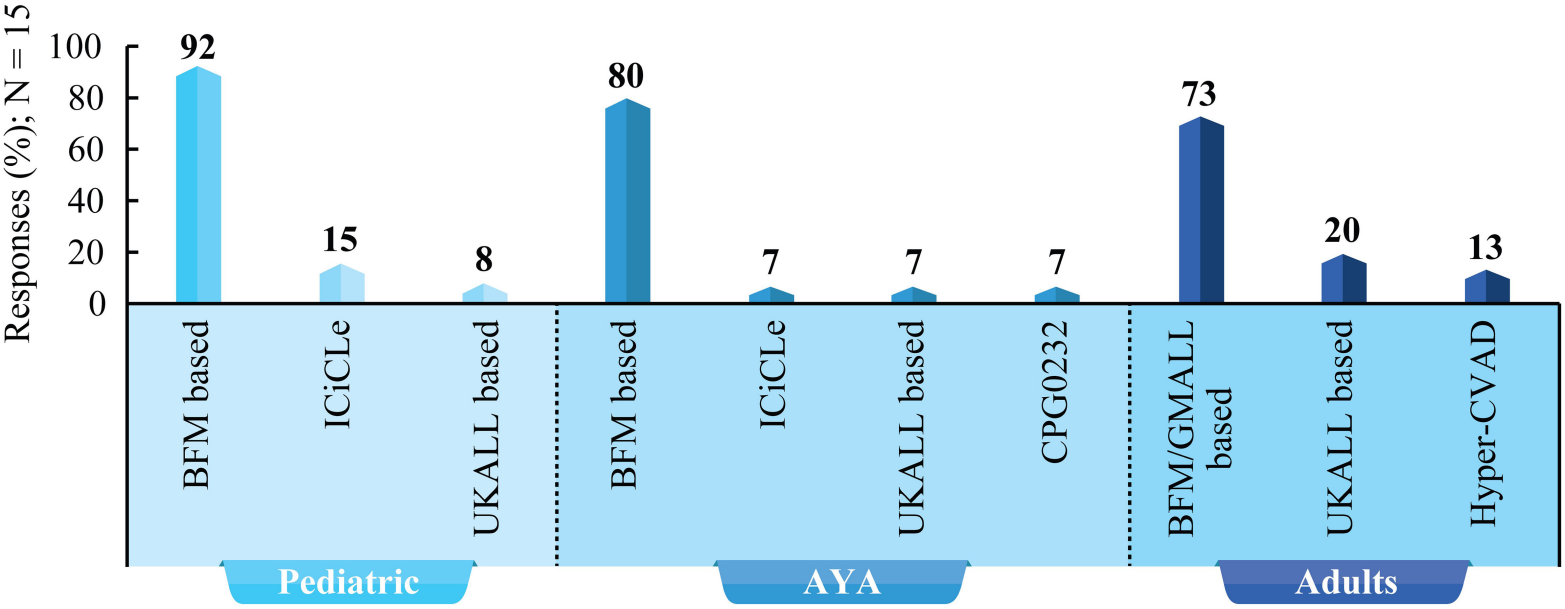
Treatment protocols for children, AYA, and adults with B-ALL: Survey results based on experts’ clinical practice. BFM, Berlin–Frankfurt–Munster; CVAD, Cyclophosphamide, vincristine sulfate, doxorubicin hydrochloride (Adriamycin), and dexamethasone; UKALL, United Kingdom Acute Lymphoblastic Leukemia; GMALL, German Multicenter Study Group for Adult Acute Lymphoblastic Leukemia; ICiCLe, The Indian Childhood Collaborative Leukemia; AYA, Adolescents and young adults.

According to the experts, age, risk stratification, comorbidities, and financial constraints are crucial factors in determining treatment strategy. Patients should be categorized into AYA and adults for the optimal choice of the treatment protocol. However, there was no consensus on the age threshold to be used in practice. Experts recommended BFM-based protocol as frontline therapy in pediatric and AYA patients with B-ALL (high consensus). BFM/GMALL-based regimen is suggested in adult patients with B-ALL (moderate consensus). **Figure 3** lists treatment protocols used in pediatric, AYA, and adult patients with B-ALL.

#### 3.2.1 MRD monitoring

The NCCN guidelines state that MRD is an essential component of patient evaluation over the course of sequential therapy (end of induction, consolidation, and surveillance) in pediatric and adult patients with ALL (14, 15). The ESMO guidelines recommend MRD monitoring to guide the decision of chemotherapy or allo-HCT after consolidation in patients with ALL (13). Furthermore, prolonged monitoring of *BCR-ABL1* MRD levels is recommended, associated with resistance mutation screening in patients with persistent MRD detection or re-increasing MRD levels (13). **Table 3** lists different methods for MRD assessment in patients with B-ALL and levels of sensitivity (24–26).

**Table 3:** Different methods for MRD assessment and level of sensitivity in patients with B-ALL.

Techniques	Sensitivity	Applicability
Flow cytometry	10^−4^	Ph− B-ALL Ph+ B-ALL
RT-PCR of *Ig/TCR* rearrangements	10^−4^–10^−5^	Ph− B-ALL Ph+ B-ALL
RT-qPCR of *BCR-ABL1* transcripts	10^−4^–10^−5^	Ph+ B-ALL
NGS of *Ig/TCR* rearrangements	10^−6^	Ph− B-ALL Ph+ B-ALL

B-ALL, B-cell lineage acute lymphoblastic leukemia; RT-PCR, Real-time reverse transcription-polymerase chain reaction; RT-qPCR, Quantitative reverse transcription PCR; Ig, Immunoglobulin; TCR, T-cell receptor; MRD, Minimal residual disease; Ph+, Philadelphia chromosome-positive; Ph−, Philadelphia chromosome-negative; NGS, Next-generation sequencing.

Adapted from: Hein K et al., 2022 (24), Abou Dalle I et al., 2020 (25), and Tierens A et al., 2021 (26).

***Consensus/recommendations on MRD assessment***

MRD is the preferred criterion for determining outcomes in patients with B-ALL. Experts recommended flow cytometry for MRD assessment in patients with B-ALL. The consensus statements on MRD monitoring in patients with B-ALL have been summarized in **Table 4**.

**Table 4:** Expert consensus/recommendations on MRD monitoring in patients with B-ALL.

**High consensus**	• Flow cytometry is indicated as the method of choice for MRD assessment. In addition to flow cytometry, RT-PCR may also be used in patients with fusion transcripts. • MRD-stratified protocols assist in decisions regarding the need and timing for allo-HCT. • In patients undergoing allo-HCT, MRD assessments should be conducted before the transplant. For Ph+ B-ALL post-allo-HCT, long-term monitoring with peripheral blood RT-qPCR can be considered once in 3 months.

B-ALL, B-cell lineage acute lymphoblastic leukemia; MRD, Minimal residual disease; RT-PCR, Real-time reverse transcription-polymerase chain reaction; allo-HCT, Allogeneic hematopoietic cell transplantation; RT-qPCR, Quantitative reverse transcription polymerase chain reaction; Ph+, Philadelphia chromosome-positive.

#### 3.2.2 CNS prophylaxis

CNS prophylaxis aims to prevent relapse or CNS disease and mainly includes intrathecal or systemic chemotherapy. Cranial irradiation is often associated with secondary neoplasms, neurocognitive dysfunction, endocrinopathy, and neurotoxicity (27). A combination of high-dose systemic therapy with CNS penetration (e.g., methotrexate or cytarabine) and intrathecal chemotherapy is quite effective, with CNS recurrence incidence being < 6% (28, 29). The NCCN recommends CNS prophylaxis to be given throughout the entire course of treatment to all patients (15, 30).

***Consensus/recommendations***

- In pediatric and AYA B-ALL patients, intrathecal methotrexate and systemic therapy is the preferred option for CNS prophylaxis (moderate consensus).
- In adult B-ALL patients, there was no consensus on the choice of therapy.

Experts agreed that CNS prophylaxis is a must in adult B-ALL patients; however, there was no consensus on the choice of therapy. Experts suggested that a combination of systemic and intrathecal chemotherapy may be considered for CNS prophylaxis in adult patients with B-ALL. The use of CNS irradiation in addition to intrathecal methotrexate may be advised based on institutional experience and infrastructure in resource-limited settings.

### 3.3 Treatment of R/R B-ALL

#### 3.3.1 Prognostic factors

The definitions for “early” and “late” relapse differ among different study groups. The BFM group study categorized time to relapse or length of first CR as (i) very early relapse (less than 18 months from diagnosis); (ii) early relapse (more than 18 months from diagnosis and less than 6 months of completion of frontline therapy); and (iii) late relapse (more than 6 months after the completion of frontline therapy) (31). In contrast, the COG defined “very early time to relapse” as the length of first CR less than 18 months from initial diagnosis; “intermediate” as 18–36 months after initial diagnosis; “early” relapse as within 36 months after initial diagnosis; or (iv) “late” relapse as 36 months or more after diagnosis (31). The ESMO and NCCN guidelines state that age (< 1 year old or ≥10 years) and white blood cell (WBC) count (50 X 10^9^ cells/L) on presentation are independent, clinically significant prognostic factors predicting lower CR rate and shorter CR duration in patients with B-ALL (13, 14). Unfavorable cytogenetics, time to relapse, site of relapse, response to first salvage therapy, performance of allo-HCT, and MRD during second CR and before allo-HCT are significant prognostic factors for survival after relapse (14, 31, 32).

***Consensus/recommendations on prognostic factors***

Experts used the BFM study group definition of “early” and “late” relapse in their clinical practice (high consensus). The expert panel agreed that the response to salvage (high consensus) and performance of allo-HCT (moderate consensus) are two key prognostic factors for CR and survival among relapsed B-ALL patients. Age, time to relapse, pre-transplant MRD negativity, donor availability, and type are other factors that need to be considered.

#### 3.3.2 Choice of therapy for R/R B-ALL

Salvage treatment after B-ALL relapse involves inducing a complete remission 2 (CR2) with intensive chemotherapy and applying consolidation, re-intensification, and maintenance therapy, or allo-HCT as a further intensification of treatment. Several studies have reported poor survival outcomes (median OS: 4.5–6 months; 5-year OS: 3%–10%) with conventional chemotherapy regimens in relapsed adult B-ALL patients (33–36). The ESMO 2016 guidelines suggest the use of new-generation TKIs, according to the results of mutational analysis of *BCR-ABL1* transcripts in patients with relapsed Ph+ ALL (13). In 2017, blinatumomab (bispecific anti-CD3/CD19 monoclonal antibody) and inotuzumab ozogamicin (InO; calicheamicin-based antibody–drug conjugate targeting CD22) received full approval from the Food and Drug Administration for R/R precursor B-ALL (Ph+ and Ph−) in adults based on promising results from phase II and phase III clinical trials (37–42). Both InO and blinatumomab have shown beneficial outcomes in terms of achieving MRD negativity (39, 43). InO treatment has shown improved rates of CR/CR with incomplete hematologic recovery and OS vs. standard chemotherapy (SC) in adult R/R ALL with high baseline disease burden (bone marrow blast [BMB] > 90%) (44). Consequently, a greater proportion of patients in the InO vs. SC arm proceeded to stem cell transplantation, irrespective of baseline BMB percentage (44).

In pediatric R/R B-ALL patients (Ph+ and Ph−), the NCCN guideline recommend (15):

- Early or late first relapse: Initial treatment with systemic therapy. If patients experience CR2 and are MRD-negative, the options are either to continue chemotherapy and receive maintenance therapy or allo-HCT. In the case of MRD-positive or if the patient experiences the first relapse after a prior allo-HCT, the options are chemotherapy, blinatumomab, CAR-T therapy, or InO before the first or second allo-HCT.
- Multiple relapses: Treatment options include chemotherapy, blinatumomab, CAR-T therapy, or InO and allo-HCT as consolidation therapy.

In Ph+ R/R B-ALL patients (AYA and adults), after *ABL1* kinase domain mutation testing, the more recent NCCN 2021 guideline recommends (14):

- TKI with or without chemotherapy followed by allo-HCT
- Blinatumomab with or without TKI followed by allo-HCT
- InO with or without bosutinib (TKI-intolerant or refractory B-ALL) followed by allo-HCT
- CAR-T therapy (in patients under 26 years with refractory B-ALL or patients with ≥ 2 relapses and failure of 2 TKIs) followed by allo-HCT.

However, in Ph− R/R B-ALL patients (AYA and adults), after MRD assessment, blinatumomab, InO, CAR-T therapy, or chemotherapy may be considered followed by allo-HCT (14). In 2018, InO received permission from Central Drugs Standard Control Organization (CDSCO), India, for the treatment of adults with R/R CD22-positive B-ALL. It is also indicated in patients with Ph+ R/R B-cell precursor ALL who have failed treatment with at least one TKI therapy (45). Currently, the CDSCO has not approved blinatumomab and CAR-T therapy for the management of R/R B-ALL except under a trial setting in India.

**Consensus/recommendations**

***Optimal choice of therapy for early or late first relapse:*** Experts favored immunotherapy (InO or blinatumomab) followed by allo-HCT for the treatment of R/R Ph+ and Ph− B-ALL patients after the first relapse (early or late; medullary/extramedullary) and to achieve MRD negativity (high consensus). The addition of TKI should always be considered for Ph+ B-ALL patients. Experts agreed that InO would be the optimal treatment of choice in adult patients with R/R B-ALL with BMB ≥ 50% if there are no resource limitations (high consensus). Concurrent use of InO with intrathecal chemotherapy was agreed upon for R/R B-ALL patients with systemic relapse and CNS disease (moderate consensus). To balance the risk of relapse against the potential risk of conditioning regimen-related toxicity, 4–6 weeks was agreed upon between the last dose of InO and allo-HCT (high consensus). Regarding the duration between the last dose of InO and the allo-HCT (where there is a need to start maintenance therapy or another chemotherapy schedule as a bridge for time to transplant), there was an agreement that if the transplant is delayed more than 6 weeks, there is a need to start such therapy as early as possible. However, no consensus was achieved regarding duration with opinions varying between 4 and 8 weeks.

***Real-world practice for early or late first relapse*:** In patients with financial constraints or prior allo-HCT at first relapse, experts recommended standard-intensive chemotherapy (with TKI for Ph+ B-ALL patients) followed by allo-HCT (high consensus). For late relapse, risk stratification and considerations for transplant would depend upon the protocol.

***Optimal choice of therapy for early or late second and subsequent relapse:*** For subsequent relapses, CAR-T therapy (if available) or palliative care (in the absence of CAR-T therapy) was suggested (early or late; medullary/extramedullary), assuming that immunotherapy has already been used in the first relapse (high consensus).

***Real-world practice for early or late second and subsequent relapse:*** No consensus was achieved for the treatment of patients with R/R B-ALL (Ph+ or Ph−) in post-transplant second or subsequent relapse.

Isolated testicular relapse is not treated differently from other relapses if it is an early relapse (high consensus). There was no consensus on whether isolated testicular relapse should be treated differently from other relapses in case of late relapse. There was a divided opinion between palliative care, low-intensity chemotherapy, and immunotherapy regarding the optimal choice of therapy for older R/R B-ALL patients (aged ≥ 60) unfit for standard-intensity chemotherapy (no consensus).

**Table 5** summarizes expert recommendations for the management of R/R B-ALL patients.

**Table 5:** Choice of therapy in relapsed/refractory B-ALL: Summary of expert consensus/recommendations.

•Optimal* choice of therapy for R/R Ph+ or Ph− B-ALL patients in the first relapse:
○ Use of immunotherapy agents (InO or blinatumomab) followed by allo-HCT is the optimal choice of therapy for R/R Ph+ or Ph− B-ALL patients in the first relapse. The addition of TKI should always be considered for Ph+ B-ALL patients. The treatment approach remains the same for early and late relapse (medullary and extramedullary) (high consensus).
○ Important determinants of allo-HCT include donor availability, depth of remission, comorbidities, and social support. Immunotherapy (preferably InO) is the recommended choice of therapy that would yield the best outcomes if offered in the first salvage (high consensus).
○ In patients with persistent residual disease, alternative treatment approaches such as immunotherapies can enhance treatment outcomes. MRD negativity has a significant impact on transplant outcomes. The choice of agent to achieve MRD negativity can be InO or blinatumomab (high consensus). Treatment with InO before transplant is associated with both improved CR and MRD negativity (moderate consensus).
○ During treatment with InO, cytoreduction is necessary for those with WBC >10,000/µL (moderate consensus). Monitoring of liver enzymes is essential during treatment with InO (high consensus).
○ Concurrent use of InO with intrathecal chemotherapy is recommended for R/R B-ALL patients with systemic relapse and CNS disease (moderate consensus).
○ The ideal period from the last dose of InO before proceeding with a transplant can be between 4 and 6 weeks. It is important to achieve a balance between preventing VOD and the risk of relapse (high consensus).
○ Conventional maintenance therapy for 2 years is recommended for patients in remission after 6 cycles of InO, who do not undergo transplant (high consensus).
•Real-world** choice of therapy for R/R Ph+ or Ph− B-ALL patients in first relapse (high consensus):
○ Consensus was reached on the use of standard-intensive chemotherapy (with TKI for Ph+ patients) followed by transplant.
○ For late relapse, risk stratification and considerations for transplant would depend upon the protocol.
•Optimal* choice of therapy for R/R Ph+ or Ph− B-ALL patients in second and subsequent relapse (high consensus):
○ CAR-T therapy is preferred if available in clinical trial settings. Palliative care is to be considered in the absence of CAR-T therapy. This is assuming that immunotherapy has already been used in the first relapse. The treatment approach remains the same for early and late relapse (medullary and extramedullary).
•Real-world**choice of therapy for R/R Ph+ or Ph− B-ALL patients in post-transplant second or subsequent relapse (no consensus):
○ For B-ALL patients with early isolated medullary relapse, responses were split between (i) palliative care and (ii) immunotherapy (InO/blinatumomab) followed by allo-HCT.
○ For B-ALL patients with early isolated extramedullary relapse, responses were split between (i) palliative care and (ii) TKI (if Ph+) and/or chemotherapy followed by allo-HCT.
○ For B-ALL patients with late relapse (both isolated medullary and isolated extramedullary), responses were split among (i) palliative care; (ii) TKI (if Ph+) and/or standard-intensive chemotherapy followed by allo-HCT; (iii) TKI (if Ph+) and/or standard-intensive chemotherapy; and (iv) immunotherapy (InO/blinatumomab) followed by allo-HCT.
•Isolated testicular relapse is not treated differently from other relapses if it is an early relapse (high consensus). There was no consensus on whether isolated testicular relapse should be treated differently from other relapses in case of late relapse.
•Optimal* choice of treatment for R/R B-ALL patients with a high disease burden:
○ InO in adult patients with BMB percentage ≥50% (high consensus).
•Real-world** choice of treatment for R/R B-ALL patients with a high disease burden:
○ Standard-intensive chemotherapy (with TKI if Ph+) (high consensus).

## 4 Discussion

The survival rates for patients with ALL have improved in the recent decade. Improvements are largely due to advances in the understanding of disease pathogenesis, molecular genetics, incorporation of MRD testing, advent of new therapeutic agents, adoption of risk-directed treatment, use of allo-HCT, and improvements in supportive care. In recent times, novel targeted immunotherapies, including monoclonal antibodies, antibody–drug conjugates, and cellular therapies, have shown significant promise in R/R settings. The biggest problem for resource-poor countries like India is devising treatment strategies that will enable patients to avail treatment at reasonable costs and obtain substantial treatment benefits. High out-of-pocket expenditures for ALL treatment and the absence of a nationwide comprehensive universal health insurance scheme are some of the biggest constraints in the management of ALL in India.

Currently, there are no country-specific guidelines/recommendations for the diagnosis and management of B-ALL from an Indian perspective. Moreover, due to the scarcity of well-designed randomized controlled trials conducted in India, oncologists rely on data from the Western world. There is a lack of consensus on the utility of treatment options in frontline and R/R settings. To the best of our knowledge, this is the first practical consensus document to guide clinicians on diagnosis, risk assessment, and treatment approach in line with the latest available evidence and guideline recommendations in Western countries. This consensus document will offer guidance to Indian hematologists/oncologists and help achieve consistency in B-ALL management across various healthcare settings.

*Strengths:* The panel members were selected to best represent the breadth of knowledge and clinical expertise in the field from all over India. There was no selection bias during the development of the expert committee.

Limitation:

- Hematopathologists were not part of the Delphi consensus panel. The panel was only limited to the clinicians with an active practice in the field.
- The patient’s voice was not included in the consensus process.
- Supportive care and follow-up are integral parts of the management of B-ALL In the questions related to the choice of therapy for R/R B-ALL, palliative care was one of the options. The panel did discuss on palliative care; however, the discussions were not elaborate.

## 5 Conclusion

In this article, we have summarized the Indian consensus on the diagnosis and management of B-ALL. Experts recommended BFM-based protocol in the front line in pediatric and AYA patients with B-ALL. BFM/GMALL-based regimen was suggested in adult patients with B-ALL. In R/R B-ALL patients with residual disease, alternative treatment approaches such as immunotherapies can enhance treatment outcomes. Immunotherapy was agreed upon as the optimal choice of therapy that would yield the best outcomes if offered in the first salvage in R/R B-ALL. InO was recommended in R/R B-ALL patients with high tumor burden and CNS relapse. In patients with financial constraints or prior transplant at first relapse (real-world practice), standard-intensive chemotherapy (with TKI for Ph+ B-ALL patients) followed by allo-HCT may be considered. For older adults, because traditional chemotherapy has been poorly tolerated, current strategies for B-ALL (both Ph+ and Ph−) rely on palliation, low-intensity chemotherapy, or immunotherapy. CAR-T therapy or palliation was suggested after transplant if patients experience recurrent relapses.

## Author contributions

All authors contributed to the article and approved the submitted version.VM contributed in the process of concept, design and moderation of the Delphi consensus activity along with drafting, review and finalization of manuscript.

## References

[1] Terwilliger T Abdul-Hay M. Acute lymphoblastic leukemia: a comprehensive review and 2017 update. Blood Cancer J (2017) 7:e577. doi: 10.1038/bcj.2017.53 28665419 PMC5520400

[2] Chiaretti S Zini G Bassan R. Diagnosis and subclassification of acute lymphoblastic leukemia. Mediterr J Hematol Infect Dis (2014) 6:e2014073. doi: 10.4084/MJHID.2014.073 25408859 PMC4235437

[3] Guru FR Muzamil J Bashir S Mahajan A. Acute lymphoblastic leukemia, the Indian scenario. MOJ Cell Sci Rep (2018) 5:33–7. doi: 10.15406/mojcsr.2018.05.00110

[4] Arora RS Arora B. Acute leukemia in children: a review of the current Indian data. South Asian J Cancer (2016) 5:155–60. doi: 10.4103/2278-330X.187591 PMC4991139 27606304

[5] Radhakrishnan VS Agrawal N Bagal B Patel I. Systematic review of the burden and treatment patterns of adult and adolescent acute lymphoblastic leukemia in India: comprehending the challenges in an emerging economy. Clin Lymphoma Myeloma Leuk. (2021) 21:e85–s98. doi: 10.1016/j.clml.2020.08.023 33189603

[6] India State-Level Disease Burden Initiative Cancer Collaborators. The burden of cancers and their variations across the states of India: the global burden of disease study 1990-2016. Lancet Oncol (2018) 19:1289–306. doi: 10.1016/S1470-2045(18)30447-9 PMC6167407 30219626

[7] de Haas V Ismaila N Advani A Arber DA Dabney RS Patel-Donelly D. Initial diagnostic work-up of acute leukemia: ASCO clinical practice guideline endorsement of the college of American pathologists and American society of hematology guideline. J Clin Oncol (2019) 37:239–53. doi: 10.1200/JCO.18.01468 PMC6338392 30523709

[8] Jain P Korula A Deshpande P Pn N Alex AA Abraham A. Adult acute lymphoblastic leukemia: limitations of intensification of therapy in a developing country. J Glob Oncol (2018) 4:1–12. doi: 10.1200/JGO.17.00014 PMC6371642 30222028

[9] Uy N Nadeau M Stahl M Zeidan AM. Inotuzumab ozogamicin in the treatment of relapsed/refractory acute b cell lymphoblastic leukemia. J Blood Med (2018) 9:67–74. doi: 10.2147/JBM.S136575 29713210 PMC5908210

[10] Dinner S Lee D Liedtke M. Current therapy and novel agents for relapsed or refractory acute lymphoblastic leukemia. Leuk Lymphoma (2014) 55:1715–24. doi: 10.3109/10428194.2013.856428 24251864

[11] Nasa P Jain R Juneja D. Delphi Methodology in healthcare research: how to decide its appropriateness. World J Methodol (2021) 11:116–29. doi: 10.5662/wjm.v11.i4.116 PMC8299905 34322364

[12] Jünger S Payne S Brearley S Ploenes V Radbruch L. Consensus building in palliative care: a Europe-wide Delphi study on common understandings and conceptual differences. J Pain Symptom Manage (2012) 44:192–205. doi: 10.1016/j.jpainsymman.2011.09.009 22704058

[13] Hoelzer D Bassan R Dombret H Fielding A Ribera JM Buske C. Acute lymphoblastic leukaemia in adult patients: ESMO clinical practice guidelines for diagnosis, treatment and follow-up. Ann Oncol (2016) 27:v69–82. doi: 10.1093/annonc/mdw025 27056999

[14] Brown PA Shah B Advani A Aoun P Boyer MV Burke PW. Acute lymphoblastic leukemia, version 2.2021, NCCN clinical practice guidelines in oncology. J Natl Compr Canc Netw (2021) 19:1079–109. doi: 10.6004/jnccn.2021.0042 34551384

[15] Brown P Inaba H Annesley C Beck J Colace S Dallas M. Pediatric acute lymphoblastic leukemia, version 2.2020, NCCN clinical practice guidelines in oncology. J Natl Compr Canc Netw (2020) 18:81–112. doi: 10.6004/jnccn.2020.0001 31910389

[16] Yeoh AE Tan D Li CK Hori H Tse E Pui C-H. Management of adult and paediatric acute lymphoblastic leukaemia in Asia: resource-stratified guidelines from the Asian oncology summit 2013. Lancet Oncol (2013) 14:e508–23. doi: 10.1016/S1470-2045(13)70452-2 PMC4059516 24176570

[17] Magrath I Shanta V Advani S Adde M Arya LS Banavali S. Treatment of acute lymphoblastic leukaemia in countries with limited resources; lessons from use of a single protocol in India over a twenty year period [corrected]. Eur J Cancer (2005) 41:1570–83. doi: 10.1016/j.ejca.2004.11.004 16026693

[18] Radhakrishnan V Gupta S Ganesan P Rajendranath R Ganesan TS Rajalekshmy KR. Acute lymphoblastic leukemia: a single center experience with Berlin, Frankfurt, and Munster-95 protocol. Indian J Med Paediatr Oncol (2015) 36(4):261–4. doi: 10.4103/0971-5851.171552 PMC4711226 26811597

[19] Trehan A Bansal D Varma N Vora A. Improving outcome of acute lymphoblastic leukemia with a simplified protocol: report from a tertiary care center in north India. Pediatr Blood Cancer (2017) 64(4). doi: 10.1002/pbc.26281 27762058

[20] Das N Banavali S Bakhshi S Trehan A Radhakrishnan V Seth R. Protocol for ICiCLe-ALL-14 (InPOG-ALL-15-01): a prospective, risk stratified, randomised, multicentre, open label, controlled therapeutic trial for newly diagnosed childhood acute lymphoblastic leukaemia in India. Trials (2022) 23(1):102. doi: 10.1186/s13063-022-06033-1 35101099 PMC8805436

[21] Malhotra P Varma S Varma N Kumari S Das R Jain S. Outcome of adult acute lymphoblastic leukemia with BFM protocol in a resource-constrained setting. Leuk Lymphoma (2007) 48:1173–8. doi: 10.1080/10428190701343255 17577781

[22] Bajel A George B Mathews V Viswabandya A Kavitha ML Srivastava A. Adult ALL: treatment outcome and prognostic factors in an Indian population using a modified German ALL (GMALL) protocol. Leukemia (2007) 21:2230–3. doi: 10.1038/sj.leu.2404785 17554379

[23] Ganesan P Jain H Bagal B Subramanian PG George B Korula A. Outcomes in adolescent and young adult acute lymphoblastic leukaemia: a report from the Indian acute leukaemia research database (INwARD) of the hematology cancer consortium (HCC). Br J Haematol (2021) 193:e1–4. doi: 10.1111/bjh.17268 PMC7610916 33656752

[24] Hein K Short N Jabbour E Yilmaz M. Clinical value of measurable residual disease in acute lymphoblastic leukemia. Blood Lymphat Cancer (2022) 12:7–16. doi: 10.2147/BLCTT.S270134 35340663 PMC8943430

[25] Abou Dalle I Jabbour E Short NJ. Evaluation and management of measurable residual disease in acute lymphoblastic leukemia. Ther Adv Hematol (2020) 11:2040620720910023. doi: 10.1177/2040620720910023 32215194 PMC7065280

[26] Tierens A Stockley TL Campbell C Fulcher J Leber B McCready E. Consensus recommendations for MRD testing in adult b-cell acute lymphoblastic leukemia in Ontario. Curr Oncol (2021) 28:1376–87. doi: 10.3390/curroncol28020131 PMC8025812 33808300

[27] Jabbour E Thomas D Cortes J Kantarjian HM O'Brien S. Central nervous system prophylaxis in adults with acute lymphoblastic leukemia: current and emerging therapies. Cancer (2010) 116(10):2290–300. doi: 10.1002/cncr.25008 20209620

[28] Sancho JM Ribera JM Oriol A Hernandez-Rivas J-M Rivas C Bethencourt C. Central nervous system recurrence in adult patients with acute lymphoblastic leukemia: frequency and prognosis in 467 patients without cranial irradiation for prophylaxis. Cancer (2006) 106:2540–6. doi: 10.1002/cncr.21948 16700036

[29] Pui CH. Central nervous system disease in acute lymphoblastic leukemia: prophylaxis and treatment. Hematol Am Soc Hematol Educ Program (2006), 142–6. doi: 10.1182/asheducation-2006.1.142 17124053

[30] NCCN guidelines for acute lymphoblastic leukemia . Available at: https://www.nccn.org/patients/guidelines/content/PDF/all-patient.pdf (Accessed 23rd September 23, 2022).

[31] Fuster JL. Current approach to relapsed acute lymphoblastic leukemia in children. World J Hematol (2014) 3:49–70. doi: 10.5315/wjh.v3.i3.49

[32] Gökbuget N Stanze D Beck J Diedrich H Horst H-A Hüttmann A. Outcome of relapsed adult lymphoblastic leukemia depends on response to salvage chemotherapy, prognostic factors, and performance of stem cell transplantation. Blood (2012) 120:2032–41. doi: 10.1182/blood-2011-12-399287 22493293

[33] Fielding AK Richards SM Chopra R Lazarus HM Litzow MR Buck G. Outcome of 609 adults after relapse of acute lymphoblastic leukemia (ALL); an MRC UKALL12/ECOG 2993 study. Blood (2007) 109:944–50. doi: 10.1182/blood-2006-05-018192 17032921

[34] Oriol A Vives S Hernández-Rivas JM Tormo M Heras I Rivas C. Outcome after relapse of acute lymphoblastic leukemia in adult patients included in four consecutive risk-adapted trials by the PETHEMA study group. Haematologica (2010) 95:589–96. doi: 10.3324/haematol.2009.014274 PMC2857188 20145276

[35] Tavernier E Boiron JM Huguet F Bradstock K Vey N Kovacsovics T. Outcome of treatment after first relapse in adults with acute lymphoblastic leukemia initially treated by the LALA-94 trial. Leukemia (2007) 21:1907–14. doi: 10.1038/sj.leu.2404824 17611565

[36] Thomas DA Kantarjian H Smith TL Koller C Cortes J O'Brien S. Primary refractory and relapsed adult acute lymphoblastic leukemia: characteristics, treatment results, and prognosis with salvage therapy. Cancer (1999) 86:1216–30. doi: 10.1002/(sici)1097-0142(19991001)86:7<1216::aid-cncr17>3.0.co;2-o 10506707

[37] Topp MS Gökbuget N Zugmaier G, Klappers P Stelljes M Neumann S. Phase II trial of the anti-CD19 bispecific T cell-engager blinatumomab shows hematologic and molecular remissions in patients with relapsed or refractory b-precursor acute lymphoblastic leukemia. J Clin Oncol (2014) 32:4134–40. doi: 10.1200/JCO.2014.56.3247 25385737

[38] Topp MS Gökbuget N Stein AS Zugmaier G Brien S Bargou RC. Safety and activity of blinatumomab for adult patients with relapsed or refractory b-precursor acute lymphoblastic leukaemia: a multicentre, single-arm, phase 2 study. Lancet Oncol (2015) 16:57–66. doi: 10.1016/S1470-2045(14)71170-2 25524800

[39] Kantarjian H Stein A Gökbuget N Wei AH Durrant S Bacon CL. Blinatumomab versus chemotherapy for advanced acute lymphoblastic leukemia. N Engl J Med (2017) 376(9):836–47. doi: 10.1080/10428194.2019.1576872 PMC5881572 28249141

[40] Kantarjian H Thomas D Jorgensen J Jabbour E Kebriaei P Rytting M. Inotuzumab ozogamicin, an anti-CD22-calecheamicin conjugate, for refractory and relapsed acute lymphocytic leukaemia: a phase 2 study. Lancet Oncol (2012) 13:403–11. doi: 10.1016/S1470-2045(11)70386-2 22357140

[41] Kantarjian HM DeAngelo DJ Stelljes M Martinelli G Liedtke M Stock W. Inotuzumab ozogamicin versus standard therapy for acute lymphoblastic leukemia. N Engl J Med (2016) 375:740–53. doi: 10.1056/NEJMoa1509277 PMC5594743 27292104

[42] Martinelli G Boissel N Chevallier P Ottmann O Gökbuget N Topp MS. Complete hematologic and molecular response in adult patients with relapsed/refractory Philadelphia chromosome-positive b-precursor acute lymphoblastic leukemia following treatment with blinatumomab: results from a phase II, single-arm, multicenter study. J Clin Oncol (2017) 35:1795–802. doi: 10.1200/JCO.2016.69.3531 28355115

[43] Jabbour E Gökbuget N Advani A Stelljes M Stock W Liedtke M. Impact of minimal residual disease status in patients with relapsed/refractory acute lymphoblastic leukemia treated with inotuzumab ozogamicin in the phase III INO-VATE trial. Leuk Res (2020) 88:106283. doi: 10.1016/j.leukres.2019.106283 31790983

[44] DeAngelo DJ Advani AS Marks DI Stelljes M Liedtke M Stock W. Inotuzumab ozogamicin for relapsed/refractory acute lymphoblastic leukemia: outcomes by disease burden. Blood Cancer J (2020) 10(8):81. doi: 10.1038/s41408-020-00345-8 32769965 PMC7414105

[45] Central drugs standard control organization (CDSCO): list of drugs imported and marketed in India . Available at: https://cdsco.gov.in/opencms/resources/UploadCDSCOWeb/2018/UploadBiologicalrDNA/jan20Form45.pdf (Accessed November 22, 2022).

