# Supplementary material for: Management of B-cell lineage acute lymphoblastic leukemia: expert opinion from an Indian panel via Delphi consensus method
Source: Front Oncol. 2023 Apr 24;13:1171568. doi: 10.3389/fonc.2023.1171568 (PMC10166232; doi:10.3389/fonc.2023.1171568)
Supplement: Supplementary file 1 [file DataSheet_1.docx]

**SUPPLEMENTARY MATERIAL**

**Table S1: Demographic and Professional Characteristics of Delphi Expert Panel**

| **Panelist no.** | **Gender** | **Age (years)** | **Zone** | **Geographic location** | **Clinical experience**  **(years)** | **Areas of expertise** | **Number of hematology patients in OPD/week** |
| --- | --- | --- | --- | --- | --- | --- | --- |
| 1 | M | 57 | South | Vellore, India | >20 | - Hematology - Acute Leukemia - Allogeneic Stem Cell Transplantation | 150 |
| 2 | F | 47 | South | Vellore, India | 15 | - Leukemia and Lymphoma | 150 |
| 3* | M | 49 | East | Kolkata, India | 19 | - Clinical Hematology - Bone Marrow Transplant - Acute Lymphocytic Leukemia | 100 |
| 4 | M | 55 | North | New Delhi, India | 23 | - Hemato-Oncology - Acute Lymphocytic Leukemia - Bone Marrow Transplant | 500 |
| 5* | F | 59 | East | Guwahati, India | 17 | - Hematology - Leukemia | 200 |
| 6** | F | 47 | West | Mumbai, India | 17 | - Adult Hematolymphoid Disease - Non-Hodgkin Lymphoma in HIV/AIDS - Adult Acute Lymphoblastic Leukemia | 1000 |
| 7 | M | 54 | North | Chandigarh, India | 26 | - Clinical Hematology Oncology - Bone Marrow Transplant - Acute Lymphocytic Leukemia | 1000 |
| 8** | M | 39 | South | Hyderabad, India | 12 | - Aplastic Anemia - Leukemia and Lymphoma - Myeloma - Stem Cell Transplantation | 200–250 |
| 9 | M | 44 | North | New Delhi, India | 15 | - Hematologic Oncologists and Bone Marrow Transplant - Acute Lymphocytic Leukemia - Allogenic and Autologous Bone Marrow Transplant for Malignant and Non-Malignant Blood Disorders, including Thalassemia and Aplastic Anemia | 100 |
| 10 | M | 43 | South | Puducherry, India | 17 | - Hematological Cancers - Supportive Care in Cancer - Acute Lymphocytic Leukemia | 150 cases of all cancers,  70–80 cases of hematological cancers |
| 11 | M | 43 | North | Delhi, India | 8 | - Acute Lymphoblastic Leukemia - Acute Myeloid Leukemia - Bone Marrow Failure - Myeloma - Adult Bone Marrow Transplant | 10–15 new acute lymphoblastic leukemia (adult plus pediatric) |
| 12 | M | 51 | West | Pune, India | 15 | - Hematology and Bone Marrow Transplant - Acute Lymphoblastic Leukemia | 100–150 |
| 13 | M | 53 | South | Bengaluru, India | >20 | - Bone Marrow Transplant (Stem Cell Transplant) - Hemato-Oncology - Lymphoma - Myeloma - Cart T cell therapy - Acute Lymphocytic Leukemia | 200–250 |
| 14** | M | 47 | West | Kolkata, India | 20 | - Hematology and Hemato-Oncology - Acute Lymphocytic Leukemia | 700 |
| 15 | M | 45 | South | Chennai, India | 23 | - Medical and Pediatric Oncology | 500 |

*Did not participate in Delphi round 2 survey.

**Did not participate in Delphi round 3 survey.

**Response Rate and Efforts Taken to Increase Participation**

In Delphi survey round 1, there was 100% participation (N=15) from the panelists. The panelists were followed up via email reminders to complete the survey. Round 2 and round 3 surveys had 87% (N=13) and 80% (N=12) participation, respectively. The response rate for each of the consensus opinions is already available within the manuscript.

**Addressing Potential Sources of Bias**

Conflicts of interest or strong personal opinions among panel members can negatively impact the reliability and validity of the process. Panel members were carefully selected, and they have wide clinical expertise and knowledge in the field. They were asked to disclose any conflicts of interest or personal biases they may have that could potentially impact their responses before the process. Anonymity was maintained during the survey process to encourage panel members to provide honest and unbiased feedback, and a third-party facilitator was used to collect and collate responses. During group discussions, all panelists were encouraged to participate actively. The differences in opinions were also discussed, and questions/statements were modified and posed for the next round of voting.

**Questionnaire Development**

An independent facilitator (BioQuest Solutions Pvt. Ltd.) was appointed for literature review and questionnaire development. The appointed Chair provided guidance on broad topics of the questionnaire and reviewed the questionnaire for its clinical relevance. The independent facilitator performed a thorough literature review to identify relevant articles between January 2001 and September 2022 using keywords such as “B-cell acute lymphoblastic leukemia,” “diagnosis,” “management,” “relapsed/refractory,” and “guidelines.” The questionnaire was broadly segmented to include relevant questions under diagnosis and risk assignment, frontline treatment, and choice of therapy in relapsed/refractory settings (optimal and real-world practice). The terms ‘optimal choice’ and ‘real-world choice’ were defined by the moderator and the experts during the first round of discussion, and the same was adopted in the second round of voting. The questionnaire was shared with the panel members through an online survey platform.

**Diversity of Panel**

A panel of 15 experts (mean age: 48.6 years; sector: public, private, and trust hospitals) was selected based on their academic track records, involvement in clinical research, and experience in the field of hematology and leukemia from all four zones of India (North, South, East, and West). This diverse panel helped to achieve a broader perspective (covering different patient profiles) and generalization of consensus.

___________________________________________________________________________________________________________________
